# Evaluation of the clinical significance of long non-coding RNA *MALAT1* genetic variants in human lung adenocarcinoma

**DOI:** 10.18632/aging.205675

**Published:** 2024-03-21

**Authors:** Shu-Hui Lin, Jeng-Wei Lu, Wang-Ting Hsieh, Ying-Erh Chou, Tzu-Cheng Su, Tun-Jen Tsai, Yun-Jung Tsai, Po-Jen Yang, Shun-Fa Yang

**Affiliations:** 1 Department of Pathology, Changhua Christian Hospital, Changhua, Taiwan; 2 Department of Medical Laboratory Science and Biotechnology, Central Taiwan University of Science and Technology, Taichung, Taiwan; 3 Department of Post-Baccalaureate Medicine, College of Medicine, National Chung Hsing University, Taichung, Taiwan; 4 Biotech Research and Innovation Centre, University of Copenhagen, Copenhagen, Denmark; 5 The Finsen Laboratory, Rigshospitalet/National University Hospital, Faculty of Health and Medical Sciences, University of Copenhagen, Copenhagen, Denmark; 6 The Affiliated High School of Tunghai University, Taichung, Taiwan; 7 Department of Occupational Therapy, Asia University, Taichung, Taiwan; 8 School of Medicine, Chung Shan Medical University, Taichung, Taiwan; 9 Translational Pathology Core Laboratory, Changhua Christian Hospital, Changhua, Taiwan; 10 Department of Family and Community Medicine, Chung Shan Medical University Hospital, Taichung, Taiwan; 11 Institute of Medicine, Chung Shan Medical University, Taichung, Taiwan; 12 Department of Medical Research, Chung Shan Medical University Hospital, Taichung, Taiwan

**Keywords:** MALAT1, single nucleotide polymorphism, EGFR, lung adenocarcinoma

## Abstract

Lung adenocarcinoma (LUAD) is the most frequent histological subtype of lung cancer, which is the most common malignant tumor and the main cause of cancer-related mortality globally. Recent reports revealed that long non-coding RNA (lncRNA) of metastasis-associated lung adenocarcinoma transcript 1 (MALAT1) plays a crucial role in tumorigenesis and metastasis development in lung cancer. However, the contribution of *MALAT1* genetic variants to the development of LUAD is unclear, especially in epidermal growth factor receptor (EGFR) mutation status. In this study, 272 LADC patients with different EGFR status were recruited to dissect the allelic discrimination of the *MALAT1* polymorphisms at rs3200401, rs619586, and rs1194338. The findings of the study showed that *MALAT1* polymorphisms rs3200401, rs619586, and rs1194338 were not associated to LUAD susceptibility; however, rs3200401 polymorphisms was significantly correlated to EGFR wild-type status and tumor stages in LUAD patients in dominant model (p=0.016). Further analyses using the datasets from The Cancer Genome Atlas (TCGA) revealed that lower *MALAT1* mRNA levels were associated with the advanced stage, and lymph node metastasis in LADC patients. In conclusion, our results showed that *MALAT1* rs3200401 polymorphisms dramatically raised the probability of LUAD development.

## INTRODUCTION

The most common cause of cancer-related death globally and a lethal malignancy, is lung cancer [1]. Non-small cell lung cancer (NSCLC) is the most often seen histological subtype of lung cancer and is largely divided into squamous cell carcinoma (LUSC) and adenocarcinoma (LUAD) [2]. Among them, LUAD accounts for about 40% of all lung cancer patients [3]. The prognosis for LUAD is still disappointing, with a 5-year survival rate of less than 20%, despite recent advancements in cancer therapeutic treatments, such as surgical resection, immunotherapy, chemotherapy, and radiation [4]. Nevertheless, once distant metastases have developed, LUAD cannot be treated surgically [5]. Consequently, based on the developing concept of precision medicine, the development of molecular pathology diagnostic technologies and tailored therapy have considerably increased the overall survival of LUAD patients [6]. Key genes involved in carcinogenesis might be thought of as therapeutic targets in precision medicine. Mutations in the epidermal growth factor receptor EGFR is one often reported molecule that is employed in the diagnosis of disease and as therapeutic targets in NSCLC [7, 8]. Curing individuals with LUAD remains difficult despite the positive clinical outcomes of molecularly targeted treatments. Thus, further research is required for the development of lung cancer treatments, particularly to identify relevant potential genetic markers [9–11].

Non-coding RNAs (ncRNAs) are further divided into two categories according to the length, less than 200 bp (Small non-coding RNAs; sncRNAs) and greater than 200 bp (Long non-coding RNAs; lncRNAs), respectively [12]. Accumulating evidence has shown that long non-coding RNAs, which have been identified as oncogenes or tumor suppressors, play crucial roles in biological processes and are involved in tumorigenesis processes, including uncontrolled proliferation, escape cell death and tumor metastasis [13–16]. Metastasis-associated lung adenocarcinoma transcript 1 (MALAT1) lncRNA, which was initially identified as an oncogene in a study of NSCLC, is situated at 11q13 [17]. Since its discovery, MALAT1 has contributed significantly to the progression, metastasis, drug resistance, and treatment of the cancer, as well as its clinical importance in predicting the tumor metastasis of early stage, particularly lung cancer [17, 18]. Subsequently, overexpression of MALAT1 was also found to be involved in tumor cell proliferation, migration, invasion and apoptosis in various cancers. In addition, increased expression level of MALAT1 can also be used as a potential biomarker for tumor diagnosis and prognosis, including liver, colorectal, pancreatic, papillary thyroid, renal cancers and gastrointestinal diffuse large B-cell lymphoma [19–24].

Many single nucleotide polymorphisms (SNPs) are linked to several cancers, according to genome-wide association studies (GWAS) [25, 26]. Recent studies have shown that genetic variations, such as those prevalent universally present in lncRNA genes, may directly or indirectly affect lncRNA expression levels in multiple ways to regulate tumor development [27, 28]. Previous studies have reported that the *MALAT1* polymorphisms were associated with cancer development, carcinogenesis and prognosis in various cancers such as hepatocellular carcinoma [29], cervical cancer [30], oral squamous cell carcinoma [31], and prostate cancer [32]. However, only a small number of researches have looked into the connection between *MALAT1* SNPs and lung cancer [33, 34]. The link between genetic variations and somatic mutations in the development of cancer has recently been studied. In NSCLC, for instance, it was discovered that SNPs were associated with EGFR mutation susceptibility [11, 35]. Furthermore, the coexistence of *MALAT1* SNPs with various EGFR mutation statuses remains unclear for the clinicopathological features of LUAD. In this study, a case-control study was conducted on the Taiwanese population to explore the relationship between *MALAT1* polymorphisms and susceptibility, and to explore the correlation between *MALAT1* SNPs and EGFR phenotypes and their impact on clinicopathological characteristics, aiming to reveal potential genetic markers affecting EGFR mutation status and prognosis in LUAD patients.

## RESULTS

A total of 272 LADC patients were included in this study, and Table 1 summarizes the characteristics of EGFR wild-type and EGFR mutation subjects recruited. The EGFR wild-type and EGFR mutation samples were split into two groups. There were 163 patients with EGFR mutation (57 males and 106 females; mean age, 65.71 + 13.50 years) compared to 109 patients (66 males and 43 females; mean age, 65.10 + 13.40 years) with the EGFR wild-type. The EGFR mutation were significantly associated with gender (p<0.001), cigarette smoking status (p<0.001), and cell differentiation (p<0.001) in relation to the clinical characteristics of these individuals. Age, tumor stage, tumor T status, clinical stage, lymph node status and distant metastasis were all similar, with no discernible differences.

**Table 1:** Demographics and clinical characteristics of 272 patients in lung adenocarcinoma with EGFR mutation status.

**Variable**	**EGFR wild type** **(N=109) n (%)**	**EGFR mutation** **(N=163) n (%)**	**p-value**
**Age**			
Mean + SD	65.10 + 13.40	65.71 + 13.50	p=0.714
**Gender**			
Male	66 (60.6%)	57 (35.0%)	p<0.001
Female	43 (39.4%)	106 (65.0%)	
**Cigarette smoking status**			
Never-smoker	49 (45.0%)	127 (77.9%)	p<0.001
Ever-smoker	60 (55.0%)	36 (22.1%)	
**stage**			
I+II	25 (22.9%)	46 (28.2%)	p=0.331
III+IV	84 (77.1%)	117 (71.8%)	
**Tumor T status**			
T1+T2	58 (53.2%)	104 (63.8%)	p=0.081
T3+T4	51 (46.8%)	59 (36.2%)	
**Lymph node status**			
Negative	28 (25.7%)	53 (32.5%)	p=0.228
Positive	81 (74.3%)	110 (67.5%)	
**Distant Metastasis**			
Negative	52 (47.7%)	78 (47.9%)	p=0.981
Positive	57 (52.3%)	85 (52.1%)	
**Cell differentiation**			
Well	8 (7.3%)	19 (11.7%)	p<0.001
Moderately	78 (71.6%)	135 (82.8%)	
Poorly	23 (21.1%)	9 (5.5%)	

Data with categories: N (%); Continuous variables: mean standard deviation (SD); The comparisons between the EGFR wild-type and EGFR mutation in LUAD were assessed using the Mann-Whitney U-test or Fisher’s exact test; Statistics were considered significant at p-values less than 0.05.

The genotype frequencies of all three SNPs (rs3200401, rs619586, and rs1194338) carrying EGFR wild-type or EGFR mutation were initially examined in LADC patients to evaluate any potential associations between *MALAT1* SNPs and the likelihood of the risk of developing EGFR mutation. The highest frequencies of these SNPs of *MALAT1* were homozygous for CC (rs3200401), AA (rs619586) and CC (rs1194338), respectively, in both EGFR wild-type or EGFR mutation groups. Statistical data demonstrated that there was no correlation between *MALAT1* SNPs and EGFR mutation (Table 2).

**Table 2:** Distribution frequency of *MALAT1* genotypes of patients with lung adenocarcinoma and multiple logistic regression analysis of EGFR mutation association.

**Genotypes**	**EGFR wild type (N=109)**	**EGFR mutation (N=163)**	**AOR (95% CI)**	**p-value**
**rs3200401**				
CC	70 (64.2%)	112 (68.7%)	1.000 (reference)	
CT	35 (32.1%)	45 (27.6%)	0.897 (0.507-1.588)	p=0.710
TT	4 (3.7%)	6 (3.7%)	0.900 (0.227-3.568)	p=0.881
CT+TT	39 (35.8%)	51 (31.3%)	0.947 (0.720-1.247)	p=0.699
**rs619586**				
AA	93 (85.3%)	140 (85.9%)	1.000 (reference)	
AG	15 (13.8%)	23 (14.1%)	1.005 (0.476-2.123)	p=0.990
GG	1 (0.9%)	0 (0.0%)	---	---
AG+GG	16 (14.7%)	23 (14.1%)	0.959 (0.664-1.384)	p=0.822
**rs1194338**				
CC	43 (39.4%)	73 (44.8%)	1.000 (reference)	
CA	47 (43.1%)	68 (41.7%)	0.898 (0.512-1.576)	p=0.708
AA	19 (17.5%)	22 (13.5%)	0.765 (0.354-1.650)	p=0.494
CA+AA	66 (60.6%)	90 (55.2%)	0.928 (0.714-1.206)	p=0.575

The AORs with 95% CIs were estimated by multiple logistic regression models after controlling for genotypes. AOR, adjusted odds ratio; CI, confidence interval.

Next, to further investigate the associations with *MALAT1* genotypes for the dominant model (CC versus CT+TT) (rs3200401) in all LADC patients of clinicopathologic characteristics such as tumor stages, tumor T status, lymph node status, distant metastasis and cell differentiation. Statistical analysis showed no association between *MALAT1* SNPs (rs3200401) and all LADC patients (Table 3). As shown in Table 4, the rs3200401 dominant model (CC versus CT+TT) was significantly associated with *MALAT1* genotypes and EGFR wild-type at tumor stages (p=0.016), but not associated with tumor T status, lymph node status, distant metastasis and cell differentiation (Table 4).

**Table 3:** *MALAT1* rs3200401 genotype distribution and clinicopathologic characteristics of lung adenocarcinoma patients.

**Variable**	**ALL (N=272)**			
	**CC (N=182)**	**CT + TT (N=90)**	**OR (95% CI)**	**p-value**
**stages**				
I+II	43 (23.6%)	28 (31.1%)	1.00	p=0.186
III+IV	139 (76.4%)	62 (68.9%)	0.685 (0.390-1.202)	
**Tumor T status**				
T1+T2	105 (57.7%)	57 (63.3%)	1.00	p=0.372
T3+T4	77 (42.3%)	33 (36.7%)	0.789 (0.469-1.328)	
**Lymph node status**				
Negative	53 (29.1%)	28 (31.1%)	1.00	p=0.736
Positive	129 (70.9%)	62 (68.9%)	0.910 (0.525-1.575)	
**Distant metastasis**				
Negative	87 (47.8%)	43 (47.8%)	1.00	p=0.997
Positive	95 (52.2%)	47 (52.2%)	1.001 (0.604-1.660)	
**Cell differentiation**				
Well/Moderately	162 (89.0%)	78 (86.7%)	1.00	p=0.572
Poorly	20 (11.0%)	12 (13.3%)	1.246 (0.580-2.678)	

The AORs with 95% CIs were estimated by multiple logistic regression models after controlling for variable. AOR, adjusted odds ratio; CI, confidence interval.

**Table 4:** *MALAT1* rs3200401 genotype distribution and clinicopathologic characteristics of EGFR wild type lung adenocarcinoma patients.

**Variable**	**EGFR wild type (N=109)**			
	**CC (N=70)**	**CT + TT (N=39)**	**OR (95% CI)**	**p-value**
**stages**				
I+II	11 (15.7%)	14 (35.9%)	1.00	**p=0.016**
III+IV	59 (84.3%)	25 (64.1%)	0.333 (0.133-0.834)	
**Tumor T status**				
T1+T2	35 (50.0%)	23 (59.0%)	1.00	p=0.368
T3+T4	35 (50.0%)	16 (41.0%)	0.696 (0.315-1.535)	
**Lymph node status**				
Negative	16 (22.9%)	12 (30.8%)	1.00	p=0.365
Positive	54 (77.1%)	27 (69.2%)	0.667 (0.277-1.607)	
**Distant metastasis**				
Negative	32 (45.7%)	20 (51.3%)	1.00	p=0.577
Positive	38 (54.3%)	19 (48.7%)	0.800 (0.365-1.753)	
**Cell differentiation**				
Well/Moderately	55 (78.6%)	31 (79.5%)	1.00	p=0.911
Poorly	15 (21.4%)	8 (20.5%)	0.946 (0.361-2.482)	

The AORs with 95% CIs were estimated by multiple logistic regression models after controlling for variable. AOR, adjusted odds ratio; CI, confidence interval.

**Figure 1:**
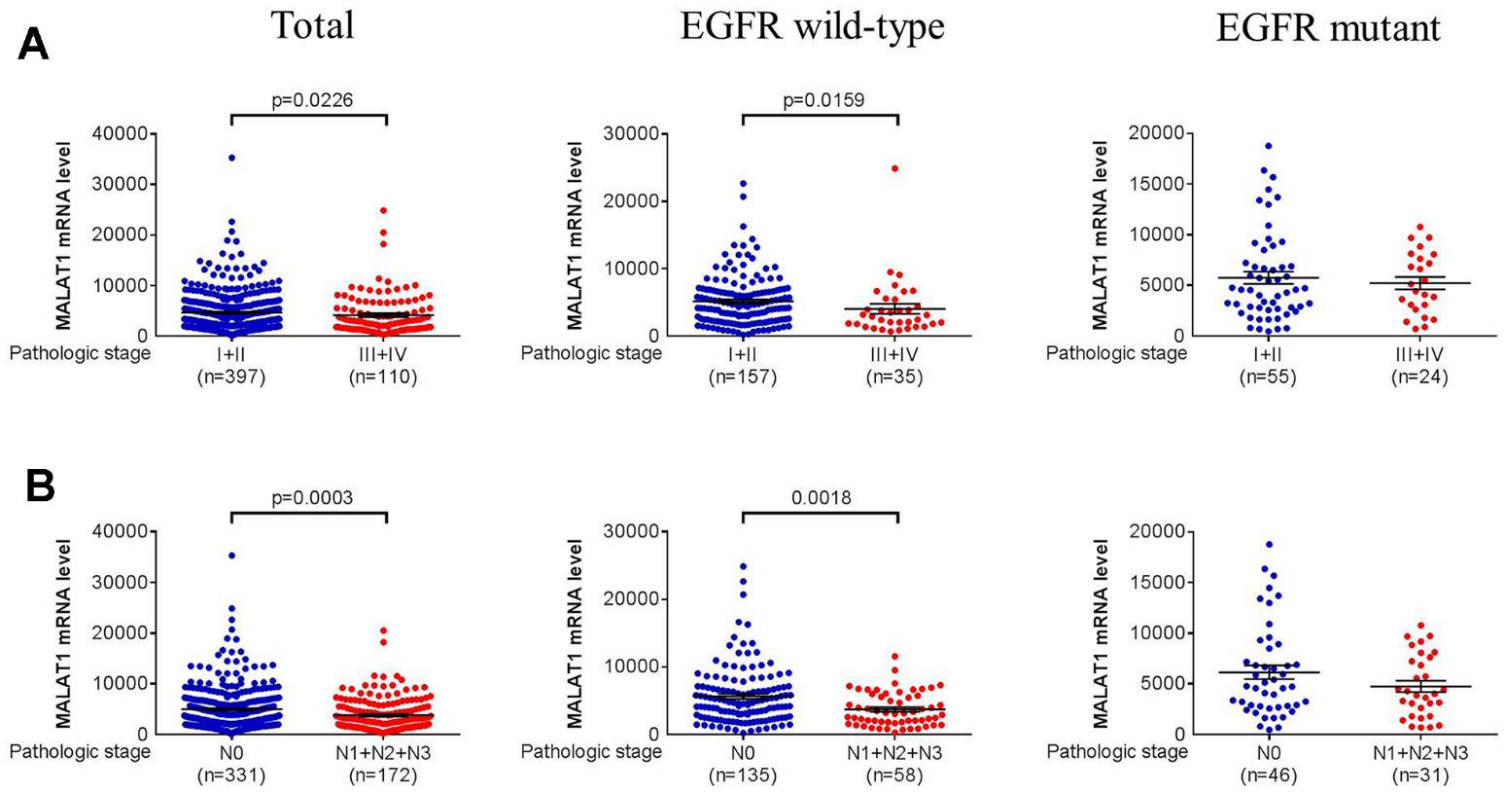
**Pathologic stage and lymph node metastases in LADC were correlated with MALAT1 mRNA expression levels.** (**A**, **B**) From the TCGA database, correlations between lower MALAT1 mRNA expression and pathologic stage or lymph node metastasis of total, EGFR wild-type and EGFR mutant in LADC. A p-value <0.05 was regarded as statistically significant using Student’s t-test.

**Figure 2:**
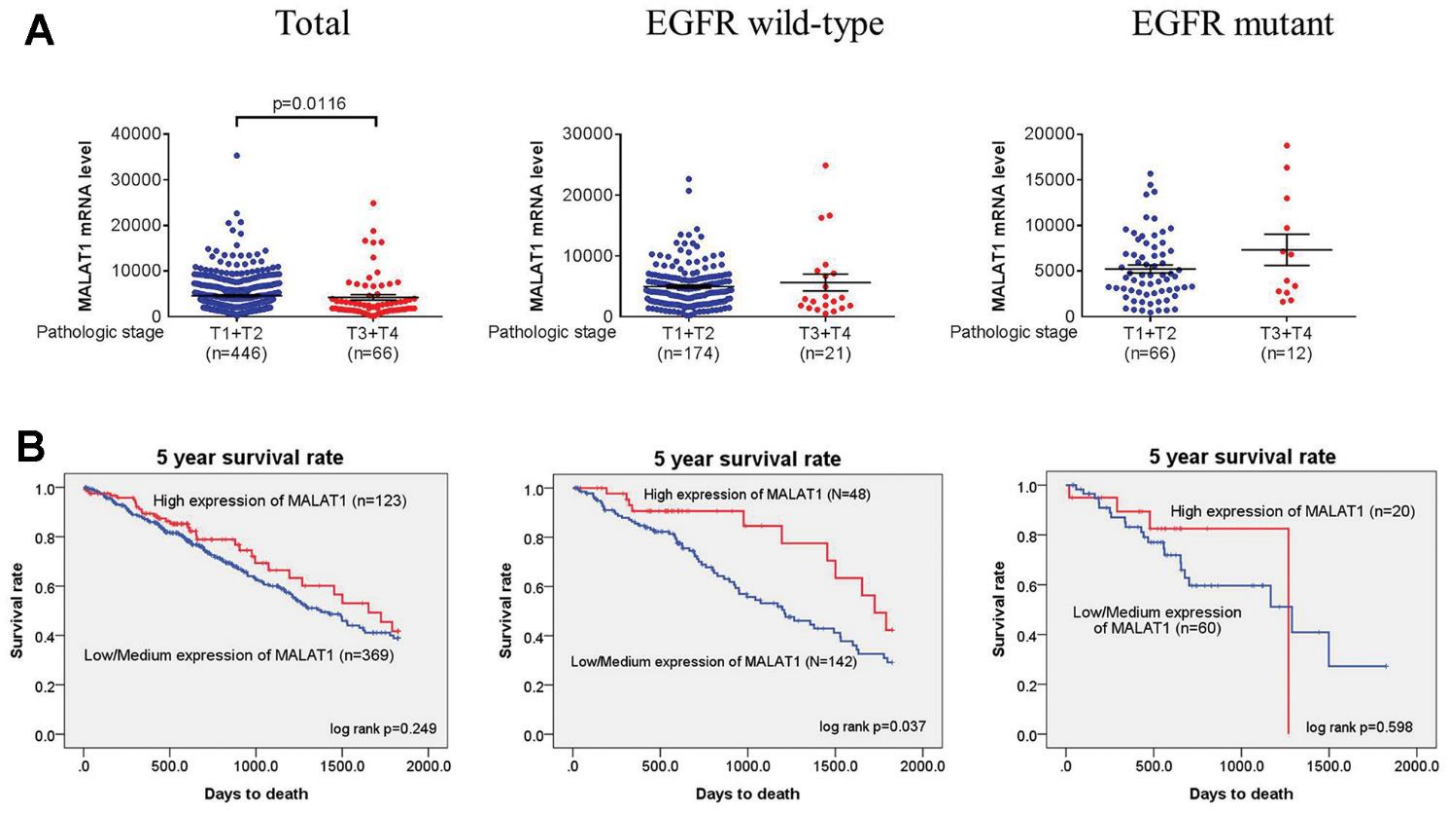
**Tumor T status in LADC was correlated with MALAT1 mRNA expression levels or 5 years survival rates.** (**A**, **B**) From the TCGA database, correlations between lower MALAT1 mRNA expression or 5 years survival rates and tumor T status of total, EGFR wild-type and EGFR mutant in LADC. A p-value <0.05 was regarded as statistically significant using Student’s t-test or log-rank test.

Further studies utilizing open-access datasets were carried out to determine the clinical significance of the MALAT1 expression in light of the genetic link between MALAT1 and LADC that was discovered. In The Cancer Genome Atlas (TCGA) dataset, we discovered that larger cases of tumors with LADC had lower MALAT1 mRNA expression levels at pathologic stage (all LADC patients: p=0.0226; EGFR wild-type patients: p=0.0159) (Figure 1A), and lymph node metastasis (all LADC patients: p=0.0003; EGFR wild-type patients: p=0.0018) (Figure 1B) in all LADC (Total) or EGFR wild-type, respectively. Moreover, LADC had lower MALAT1 mRNA expression levels at large tumor T status (p=0.0116) in all LADC patients (Figure 2A). To elucidate the prognostic role of MALAT1 in patients with LADC, the 5 years survival rates were estimated using Kaplan-Meier survival curves from all LADC (Total), EGFR wild-type and EGFR mutation, respectively. The log-rank test further confirmed that patients with low expression of MALAT1 had a significantly worse prognosis than patients with high expression of EGFR wild-type in LADC patients (Figure 2B).

## DISCUSSION

MALAT1 lncRNA has recently been discovered to have a significant part in an expanding spectrum of illnesses and their progressions, including diabetes and its related complications [36, 37], inflammatory disorders such as Coronavirus disease 2019 (COVID-19) [38, 39], and sepsis, in addition to its well-known function in the progression of cancers [40, 41]. A pleiotropic role for MALAT1 in controlling gene expression and signaling pathways under different pathophysiological situations is suggested by the fact that it is ubiquitously linked with genomes in the areas crucial for transcriptional regulation as well as RNA processing [42]. As a result of its abundance and abnormal expression, MALAT1 lncRNA plays a critical role in the development of several malignancies, including lung cancer colorectal cancer, and bladder cancer [19, 43, 44]. According to reports, elevated MALAT1 expression is associated with a poor prognosis, tumor development, metastasis, chemoresistance, and tumor radioresistance [19, 45, 46].

According to evidence, structural changes caused by genetic variations in lncRNA may affect how it expresses or functions, which might participate in tumor development [47]. In recent years, studies have also revealed that genetic polymorphisms of *MALAT1* have susceptibility to cancers [31, 32, 40]. In our study, the cohort of LUAD demographics and fundamental traits were comparable to those of previous Asian cohort [48, 49]; these cohort also showed that LUAD patients with an EGFR mutation were more likely to be female, never-smokers, and to have better cell differentiation than the EGFR wild-type group. The absence of smoking was linked to the development of LUAD, whereas EGFR mutations were more common among never-smoker (Table 1). Nevertheless, two researches showed that the *MALAT1* SNPs (rs619586; A/G) dramatically decreased the risk of lung cancer, and the SNPs (rs3200401; CT) was linked to the susceptibility to NSCLC and lung squamous cell carcinoma (LSCC) in Chinese population [33, 34]. Moreover, previous study showed that the rs3200401C allele of the *MALAT1* polymorphism could be a protective factor for cervical cancer development [50]. According to our findings, this is the first study to investigate relationships between *MALAT1* SNPs (rs3200401, rs619586, and rs1194338) and the presence of EGFR mutations as well as clinicopathologic characteristics at a Taiwanese population in LUAD (Table 2). We provide a novel discovery that LUAD patients with tumor stages harboring *MALAT1* (rs3200401; CC and CT+TT) heterozygotes had a significantly associated with EGFR wild-type (Table 4), but not in all LADC patients (Table 3).

The ability of certain lncRNAs to act as tumor suppressors or oncogenes, which are critical in the regulation of cell growth, division, and differentiation, has been demonstrated in earlier studies. These lncRNAs also employ multiple mechanisms to regulate cancer status, and the expression of lncRNAs may contribute to cancer initiation and progression [34]. MALAT1 was shown to be an abundant nuclear-enriched transcript that is expressed in healthy organs such the pancreas, lung, and nervous system. Elevated MALAT1 highly expression has been detected in lung cancer, endometrial stromal sarcoma, hepatocellular carcinoma, tongue squamous cell carcinoma, breast cancer and pancreatic cancer [34, 51, 52]. A study finds that lncRNA-MALAT1 is involved in NSCLC progression by targeting miR-202 [53]. Additionally, MALAT1 has also been used as a biomarker for lung cancer metastasis using a loss-of-function model demonstrates that MALAT1 has an active role in regulating gene expression that is responsible for preventing lung cancer metastasis [43]. High expression of MALAT1 promoted the progression of NSCLC through the extracellular signal-regulated kinase 1/2 (ERK)/mitogen-activated protein kinase (MAPK) signaling pathway [54].

In our study, we further investigated that clinical significance of the mRNA expression in light of the genetic link between MALAT1 and pathologic stage at EGFR wild-type and EGFR mutation in LADC. Lower expression mRNA levels of MALAT1 were associated with pathologic stage (Figure 1A) and lymph node metastasis (Figure 1B) and tumor T status in all LADC or EGFR wild-type. Intriguingly, the TCGA study revealed that patients with LADC who had low expression of MALAT1 had a considerably poorer prognosis than those who had high expression of EGFR wild-type (Figure 2B). Nevertheless, previous studies have shown that MALAT1 functions as an oncogene in cancer [55, 56]; however, our data suggest that MALAT1 may have a tumor suppressor function in lung cancer cells which is consistent with previous result [33, 57].

While interpreting our findings, it is important to consider the present study of limitations. In this study, the sample size of patients was still insufficient which may have limited statistical impact on the accuracy and precision of the results and a larger independent cohort was required to further confirm the influence of *MALAT1* SNPs on EGFR mutation susceptibility and LUAD development. In addition, this study is limited to the Taiwanese population and other ethnic groups still need to be compared with the current results. Additionally, the relationship between *MALAT1* genetic variations and its level of expression in LUAD cannot be supported by our research. Consequently, it is necessary to collect both mRNA and DNA from the same sample and to further confirm this issue in future research.

In summary, our results provide evidence that in Taiwanese population, the polymorphisms rs3200401 in *MALAT1* was associated with the risk and low expression of MALAT1 had a significantly worse prognosis of EGFR wild-type in LUAD. It will need further research to confirm or disprove the findings of this preliminary investigation.

## MATERIALS AND METHODS

### Study subjects and sample collection

A total of 272 patients with lung adenocarcinoma were recruited for the present study, either with or without an EGFR mutation. For each patient, the medical records were consulted to determine their age, gender, cigarette smoking status, tumor stage, tumor T status, lymph node status, distant metastasis and cell differentiation. Clinical staging was determined using the TNM staging approach according to the seventh edition of the American Joint Committee on Cancer (AJCC) Staging Manual for each patient. After gaining the fully informed permission of all participants, the study proposal was approved by the Institutional Review Board (IRB) at Chung Shan Medical University Hospital (IRB No. CS1-20144).

### Genomic *MALAT1* SNPs detected from peripheral blood in LUAD patients

Genomic DNA was extracted using a QIAamp DNA blood micro kit from peripheral blood samples of the research participants (Qiagen, Valencia, CA, USA). The allelic discrimination of these three *MALAT1* SNPs including rs3200401 (assay ID: C_3246069_10), rs619586 (assay ID: C_1060479_10), and rs1194338 (assay ID: C_11661801_10) was evaluated by utilizing the TaqMan SNP Genotyping Assay by using the ABI StepOnePlusTM Real-Time PCR System (Applied Biosystems, Foster City, CA, USA). ABI SDS version 3.0 software was used to compute the allelic frequency [9, 29].

### Statistical analysis

The multiple logistic regression methods were used to calculate the genotype with LUAD risk correlation while controlling for relevant variables. The multiple logistic regression models were used to evaluate adjusted odds ratios (AORs) and 95% confidence intervals (CIs). Using the Mann-Whitney U test or Fisher’s exact test, significant differences in demographic data between EGFR mutation and EGFR wild-type controls were assessed in LUAD patients. For the potential association between MALAT1 expression and clinical status of LUAD, we use the data of lung adenocarcinoma obtained from The Cancer Genome Atlas (TCGA) to analyze this issue [58]. Furthermore, the cumulative survival rates were determined using the log-rank test, and the Student’s t-test was used to compare the MALAT1 expression levels. The analyses were performed using Statistical Product and Service Solutions (SPSS, version 17) (SPSS, Inc., Chicago, IL, USA). A p-value <0.05 was regarded as statistically significant.

### Data availability statement

The data used to support the findings of the present study are available from the corresponding author upon request.
